# Supplementary material for: Splice-Junction-Based Mapping of Alternative Isoforms in the Human Proteome
Source: Cell Rep. Author manuscript; Available in PMC 2020 Jan 15. (PMC6961840; doi:10.1016/j.celrep.2019.11.026)
Supplement: 2 [file NIHMS1546469-supplement-2.zip › DF1/PXD010154/Heart/Heart_81_RPL14_RQTSIQNGQPHDGPR.pdf]

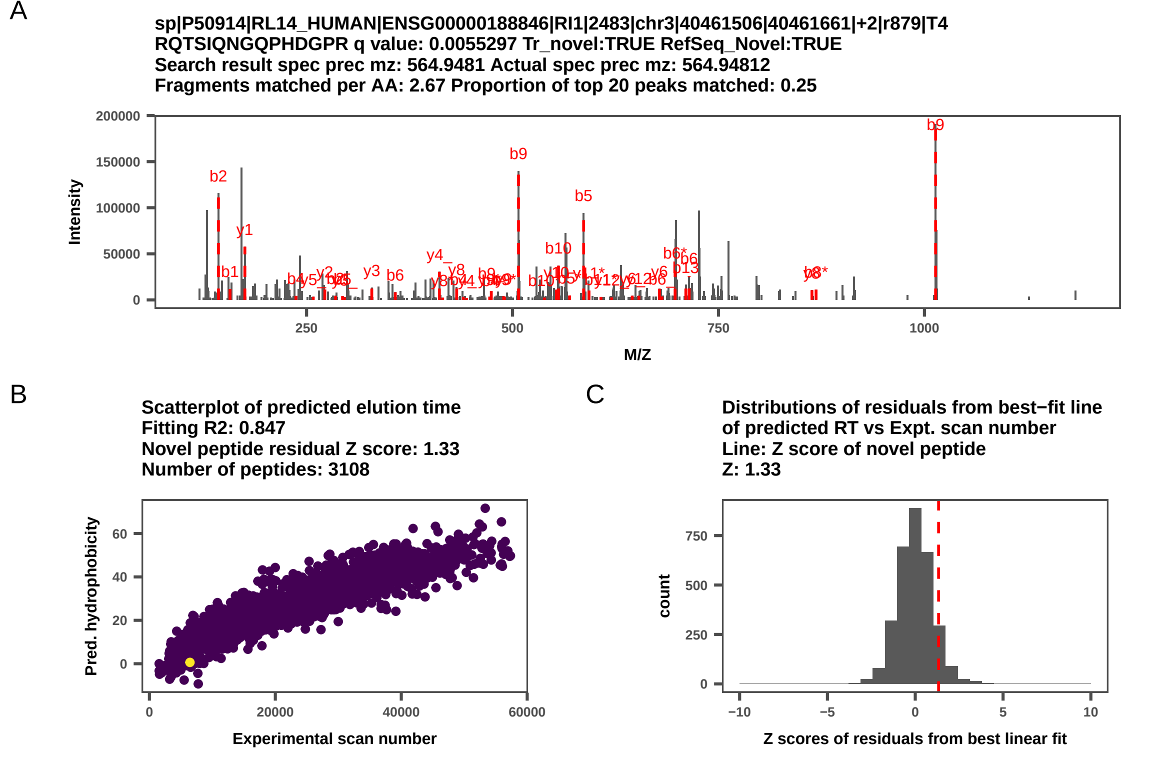
A
sp|P50914|RL14_HUMAN|ENSG00000188846|RI1|2483|chr3|40461506|40461661|+2|r879|T4
RQTSIQNGQPHDGPR q value: 0.0055297 Tr_novel:TRUE RefSeq_Novel:TRUE
Search result spec prec mz: 564.9481 Actual spec prec mz: 564.94812
Fragments matched per AA: 2.67 Proportion of top 20 peaks matched: 0.25
Intensity
200000
150000
100000
50000
0
250
500
750
1000
M/Z
b2
y1
b1
b9
b5
b10
y3
b6
y8
b13
b9
B
Scatterplot of predicted elution time
Fitting R2: 0.847
Novel peptide residual Z score: 1.33
Number of peptides: 3108
Pred. hydrophobicity
60
40
20
0
0
20000
40000
60000
Experimental scan number
C
Distributions of residuals from best−fit line
of predicted RT vs Expt. scan number
Line: Z score of novel peptide
Z: 1.33
count
750
500
250
0
−10
−5
0
5
10
Z scores of residuals from best linear fit
